# Developed wearable miniature sensor to diagnose initial perturbations of cardiorespiratory system

**DOI:** 10.1049/htl.2018.5027

**Published:** 2018-11-15

**Authors:** Reza Abbasi-Kesbi, Alireza Nikfarjam, Ardalan Akhavan Hezaveh

**Affiliations:** 1 Faculty of New Sciences and Technologies, MEMS & NEMS Laboratory, University of Tehran, Tehran, Iran; 2 Department of Biomedical Engineering, Faculty of Engineering, Science and Research Branch of Tehran, Islamic Azad University, Tehran, Iran

**Keywords:** patient monitoring, cardiovascular system, electrocardiography, pneumodynamics, bioMEMS, microsensors, biomedical equipment, microfabrication, patient diagnosis, biomedical systems, miniature wireless sensor, respiration rate, cardiorespiratory system, electrocardiogram sensor, belt sensor, cardiovascular system problems, initial perturbations, microelectromechanical systems, miniature motion sensors, heartbeat, wearable miniature sensor, chest movements, gold standard, root-mean-square errors, standard deviation, in-home health monitoring

## Abstract

The progress of microelectromechanical systems tends to fabricate miniature motion sensors that can be used for various purposes of biomedical systems, particularly on-body applications. A miniature wireless sensor is developed that not only monitors heartbeat and respiration rate based on chest movements but also identifies initial problems in the cardiorespiratory system, presenting a healthy measure defined based on height and length of the normal distribution of respiration rate and heartbeat. The obtained results of various tests are compared with two commercial sensors consisting of electrocardiogram sensor as well as belt sensor of respiration rate as a reference (gold standard), showing that the root-mean-square errors obtain <2.27 beats/min for a heartbeat and 0.93 breaths/min for respiration rate. In addition, the standard deviation of the errors reaches <1.26 and 0.63 for heartbeat and respiration rates, separately. According to the outcome results, the sensor can be considered an appropriate candidate for in-home health monitoring, particularly early detection of cardiovascular system problems.

## 1 Introduction

Today, most of older adults suffer from health problems, and continuous monitoring of the patient health is mandatory to guarantee safety of social life and prevent the injuries [1, 2]. Currently, the deaths of the cardiovascular diseases are rapidly increasing, and according to this the cardiorespiratory signal monitoring accounts for a vital sign to assess individuals’ health [3]. Although there are ample devices in hospitals or clinics to monitor cardiorespiratory system, the patients suffer from wasting time and cost for attending the places, especially on a long trip of the remote area [4]. Furthermore, the crowded hospitals and clinics have made problems for the patients when referring to the place to meet health professionals for the needed treatment and their routine clinical checkups [5]. Currently, the progress of telecommunications and information technologies has created a new concept as telemedicine to decline the drawbacks at the medical emergency [6]. The telemedicine proposes various location-based services to the patients at home during daily life [7]. The vital sign of the patient can be recorded in a smart home by employing the latest technologies in the wearable sensor. The data can be transferred via the Internet to medical databases to investigate the healthcare professionals [8].

Wearable monitoring systems were offered in the late 1990s [6]. There are several kinds of wearable sensors in the market for cardiorespiratory system monitoring that their common ones are electrocardiogram (ECG) [8], a phonocardiogram (PCG) [9] and seismocardiogram (SCG) [10]. The ECG is the process of recording the electrical signal of heart over a period of time by putting electrodes on the patient's chest. Owing to the shocking movements of the heart, ECG fails to trace the instantaneous mechanical state of the heart, consequently suffers from movement artefact [11]. Another essential drawback is an imperfect temporal resolution in cardiac imaging that can cause stress in patients during the long data processing time [12]. Other systems, namely PCG, work based on recordings of the sounds made by the heart during a cardiac cycle. The PCG systems are mainly generated by the blood flow and consequently do not cover the infrasonic range, namely frequencies below 25 Hz [13]. Recently, technological advances in miniature biosensing devices, smart textiles, microelectronics and microelectromechnical systems (MEMS) have propelled patients’ health monitoring by proposing SCG systems. The SCG systems measure the produced accelerations of the chest wall by a myocardial movement, earlier known as ballistocardiogram [8]. The vibration is recorded by placing an MEMS accelerometer on the sternum, while the patient lies supine [10]. The SCG systems have been recently recommended for healthcare at home instead of ECG and PCG due to being comfortable, non-bulky and non-intrusive.

In this Letter, a small wireless sensor for heartbeat and respiration rate monitoring based on chest movements (or SCG) is developed. The component of the developed sensor consists of a microcontroller, an accelerometer as well as a transceiver that all of them are attached to a printed circuit board (PCB) benefitting from low-profile and low-weight features compared with other methods in [10, 14, 15]. In comparison to another SCG system in [10, 14, 15], the proposed system can accurately estimate heartbeat and respiration rate. Therefore, the root-mean-square error (RMSE) of the proposed system are obtained as 2.27 for heartbeat and 0.93 for respiration rate. Moreover, the system is able to estimate the initial problems of the cardiorespiratory system, while the other works in [10, 14, 15] do not have this advantage.

## 2 Proposed mechanism

The methodology of the Letter is divided into two sections: measurement and separation of heartbeat and respiration rate using a developed sensor and then presenting a healthy measure for estimating initial problems in the cardiorespiratory system.

### 2.1 Heartbeat and respiration rate measurement

**Fig. 1:**
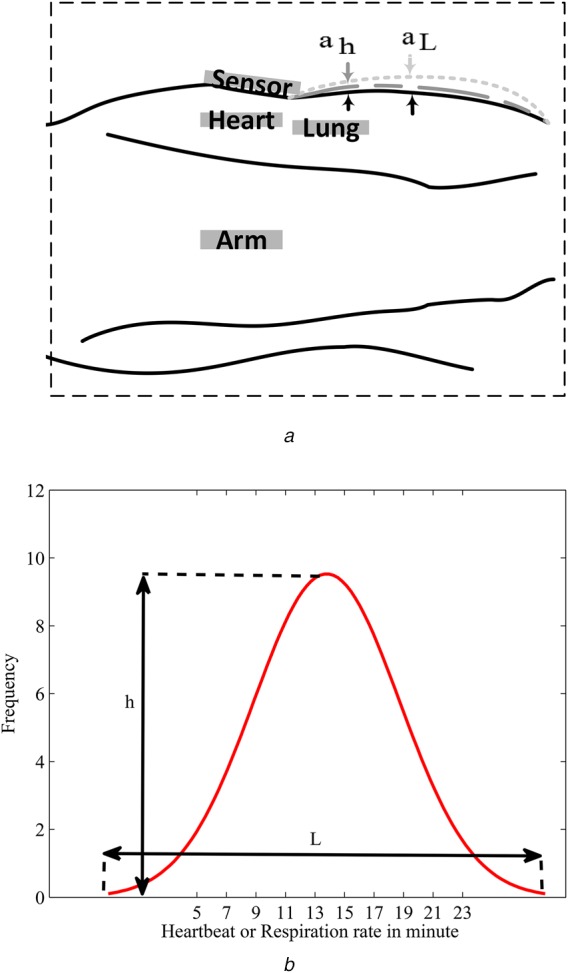
Proposed mechanism *a* Measurement of heartbeat and respiration rate *b* Diagnosis of perturbation in the cardiorespiratory system

A cardiorespiratory system can cause small accelerations in the chest and abdomen. The acceleration can be divided into two separate short- and long-term accelerations, which are considered as indicators for heartbeat and respiration rate, respectively. The acceleration due to lung activity denoted by $a_{\rm L}$ is larger with slower frequency while a higher frequency that is the result of the heart's muscle activity is denoted by $a_{\rm H}$ (Fig. 1*a*). The total acceleration can be defined as the equation below:

(1) $$a_{\rm T} = a_{\rm L} + a_{\rm H}\eqno\lpar 1\rpar $$

To obtain the variations, a motion sensor to measure both heartbeat and respiration is used which can record both short- and long-term accelerations of a patient's chest. To this end, an accelerometer can be proposed. Also, using a digital filter is mandatory to de-noise and also separate these two accelerations from together. To this end, a high-pass and low-pass Butterworth filter are designed to obtain heartbeat and respiration rate from the chest accelerations. The Butterworth filters are a second-order high-pass digital filter with the sampling rate of 50 Hz and a cut-off frequency of 0.5 Hz, which corresponds to a normalised value of 0.01.

### 2.2 Healthy measure

To identify problems in a cardiorespiratory system, first, a lot of data are collected from the lung activities of different persons. Then, a normal distribution of the data is plotted (Fig. 1*b*). By having the length (*L*) and height (*h*) of the plots, a healthy measure of respiration rate as (2) is defined. This method is reported for the heartbeat and its healthy parameter is obtained as the equation below:

(2) $$h_{{\rm rr}} = \displaystyle{{L_{{\rm hr}}} \over {h_{{\rm hr}}}}\eqno\lpar 2\rpar $$

(3) $$h_{{\rm hr}} = \displaystyle{{L_{{\rm rr}}} \over {h_{{\rm rr}}}}\eqno\lpar 3\rpar $$

## 3 Components of the developed system

**Fig. 2:**
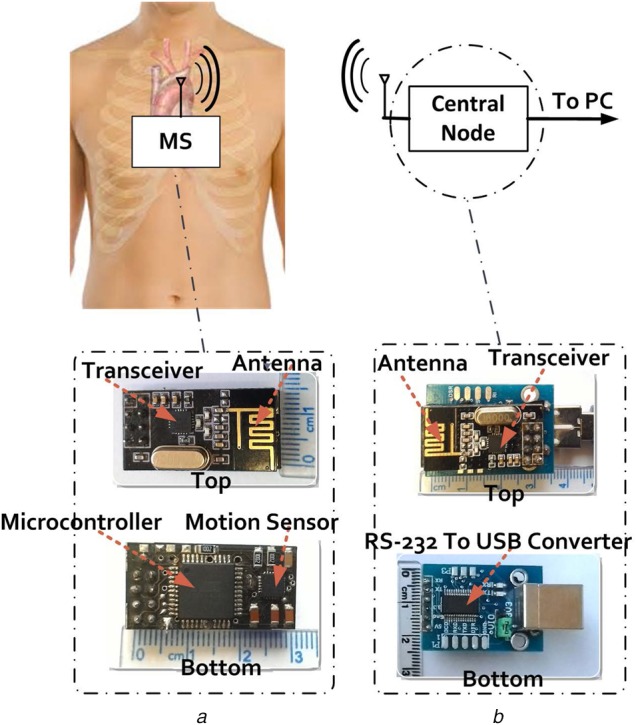
Measurement setup for recording heartbeat and respiration rate *a* Prototype of the proposed sensor *b* Developed circuit for the central node

The measurement system for estimating the heartbeat and respiration rate consists of a developed sensor (sensor node) and a central node for transmitting the measured data to a personal computer (PC) for displaying the data as shown in Fig. 2. Similar to a typical wireless sensor [16, 17], the proposed system consists of an accelerometer sensor, a microcontroller (Alf and Vegard's RISC processor (AVR) 32 bit microcontrollers), and a transceiver which operates at industrial, scientific and medical band at the frequency of 2.45 GHz (Fig. 2*a*) as well as its output power and bit rate are set at 0 dBm and 1.8 Mbps, respectively. The employed transceiver has features such as low-power consumption, high bit rate, high reliability of data transmission and low-cost. The digitised data of the accelerometer sensor are received by the microcontroller and then are sent to the transceiver for transmitting wirelessly to the central node. All the components of the proposed sensor were embedded on a small PCB and supplied with a 3.7 V_DC_ by a lithium polymer battery. Also, the central node consists of a transceiver, a processor as well as a universal serial bus (USB) to transistor-transistor logic (TTL) convertor (Fig. 2*b*). The obtained data are gotten on the air, using the transceiver in the central node and then are sent to the microcontroller, which communicates with a PC through serial port. It should be noted that the mentioned components were mounted on another PCB that its voltage was supplied from the USB port. Furthermore, the computer was used to store the data with a sampling rate of 50 sample/s.

Furthermore, other features can be mentioned for the sensor that highlights it than previous works. As it can be seen in Fig. 2, the size of the sensor is 16.5 × 29.1 × 8.5 mm^3^ that in comparison with other works in [10, 14, 15], the developed sensor benefits less size. Also, low-weighting (20 g) allows users to carry it easily, being portable. Another specification of the sensor is low-power consumption (60 mW). So, it can be supplied with a 3.7 V lithium polymer battery 180 mA for 10 h.

## 4 Results and discussion

Statistically, death toll associated with the cardiorespiratory system is dramatically enhancing, particularly during the sleep period. Thus, using a continuous monitoring of patient health can be effective and a sensor, which can accurately monitor respiration and heartbeat in-home, plays a pivotal role in reducing the toll.

### 4.1 Accuracy investigation of the developed sensor

To obtain precision of the developed sensor, a volunteer is asked to wear the sensor on the place between chest and abdomen as shown in Fig. 2. Then, an ECG apparatus similar to [18] was mounted beside the proposed sensor on the chest to compare heartbeat of the developed sensor with a gold standard. The commercial ECG device is a 1-lead ECG that measures data of ECG while it is connected to the PC through the USB port for evaluation of the measured information. Additionally, a commercial belt of respiration rate around the abdomen similar to [19] is used as a gold standard to evaluate the accuracy of the proposed sensor in measuring respiration rate.

**Fig. 3:**
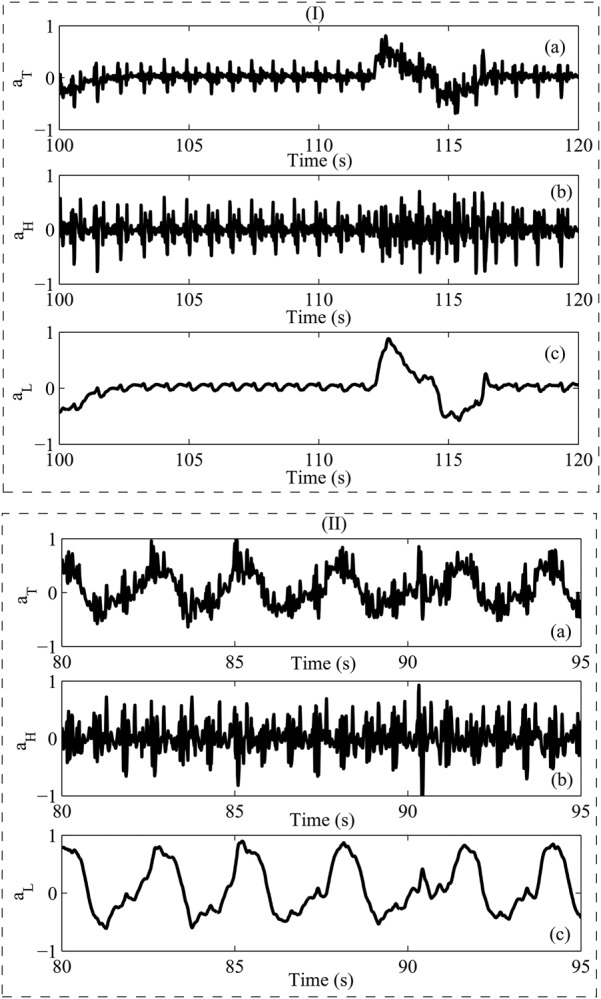
Snapshot of the obtained results of the developed sensor for two tests including (I) holding breath and (II) rest *a* Raw data *b* Heartbeat and *c* Respiration rate

To determine the accuracy of the proposed sensor, two scenarios are considered. The first scenario is performed by making the volunteer to lie down who is asked to hold his breaths and take a deep breath after a while and its results are shown in Fig. 3*a*. As the results illustrate, respiration rate obtained 5 breaths/min while heartbeat was obtained 68 beats/min. This test is done for about 600 s. In the second scenario, the volunteer is requested to lie down and the developed sensor records the heartbeat and respiration rates of about 600 s while he rests. The obtained results show the volunteer's heartbeat is 76 beats/min while the respiration rate is 20 breaths/min (Fig. 3*b*). One of the important results obtained of the two experiments is that heartbeat decreases when the volunteer holds breath in the rest of the test.

**Fig. 4:**
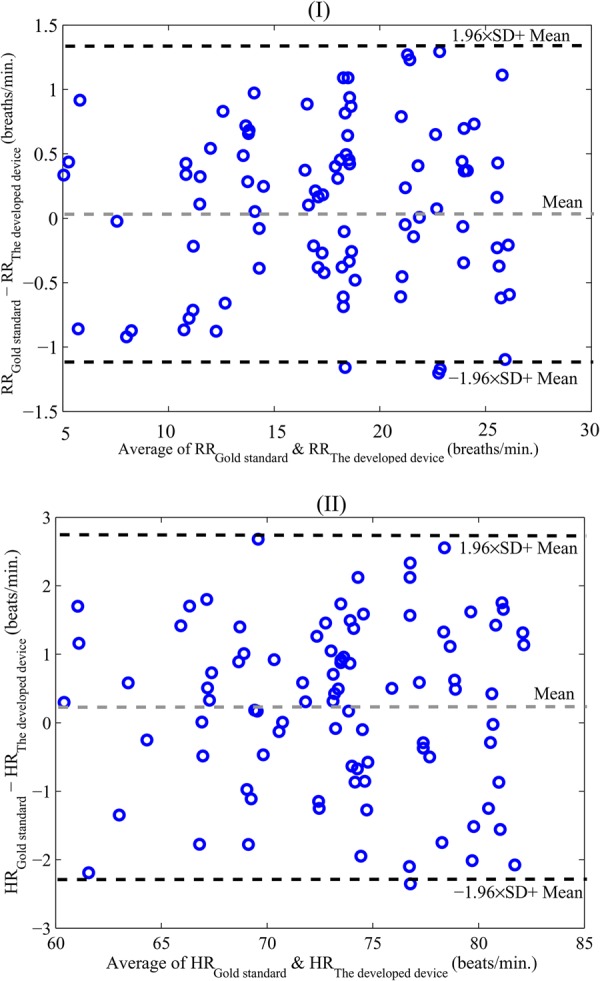
Bland–Altman plots *a* Respiration rate *b* Heartbeat measurements. The graphs show the agreement of 90 pairs of measurements. Mean error is shown with slashed light grey and 95% limits are shown with slashed dark grey lines

Bland–Altman analysis [20, 21] is considered for accuracy investigation of the obtained results from the developed sensor. Fig. 4*a* illustrates a Bland–Altman plot of the respiration rate obtained from two scenarios (rest and holding breath) for the 90 pairs of measurements in the presented sensor and gold standard system. This plot is obtained by the difference and average of the respiration rate from the obtained results of the proposed sensor and the gold standard. As the results reveal, the mean error is 0.1 breaths/min with 95% limits of agreement −1.15 to 1.34 breaths/min. Also, Bland–Altman plot of the heartbeat from the two scenarios for the 90 pairs of measurements have been shown in Fig. 4*b*. In this case, the mean error is 0.24 beats/min with 95% limits of agreement −2.2 to 2.8 beats/min. Additionally, the plot gives valuable data such as RMSE and SD of the results that were obtained 1.26, 0.63 breaths/min for respiration rate measurements and also 2.27, 0.93 beats/min for heartbeat measurements, respectively (Table 1). Accordingly, a very good agreement was obtained between the proposed system and the ECG in [18] and respiration system in [19].

**Table 1:** Error of the developed sensor (number in minutes)

Static parameters	Heartbeat	Respiration rate
RMSE	0.93	2.27
SD	0.63	1.26

### 4.2 Diagnosis of cardiorespiratory system perturbations

**Fig. 5:**
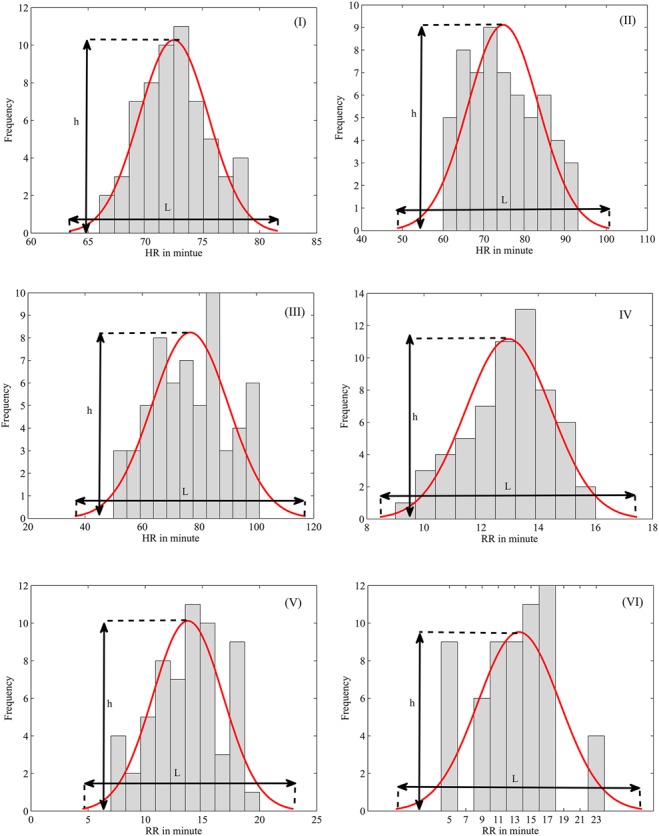
Normal distribution of the obtained results for a heartbeat *a* Healthy person *b, c* Unhealthy person. The normal distribution of the obtained results for respiration rate *d* Healthy person *e*, *f* Unhealthy person

**Fig. 6:**
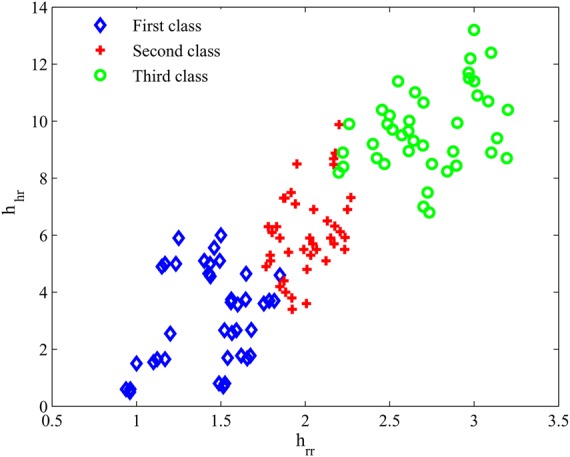
Border of healthy and unhealthy cardiorespiratory system using (2) than (3) for 30 persons from every class

To identify the problems in the cardiorespiratory system, the data of the respiration rate and heartbeat are recorded for about 1 h from three classes consisting of healthy persons (first class), persons are entering to emergency conditions (second class) and persons have dangerous conditions (third class). Data of the first class were collected at home, whereas for second class we must refer to the doctor's surgery. However, we went to an intensive care unit for recording data from the third class. Although we had an ordinary environment (with ambient noise and temperature and humidity that could change) for recording the data from the first and second classes, the environment of third class was very special (very quiet and 25 C and low humidity) for maintaining patient. All of the persons rest while their respiration rate and heartbeat are collected by the developed sensor as well as ECG set in [18] and respiration system in [19]. The normal distribution of the obtained data was calculated and shown in Fig. 5 for three classes. As Fig. 5*a* shows, the length of the normal distribution of the heartbeat of a healthy person is 17 while its height is 10. By (3), the healthy measure for the heartbeat is obtained as 1.7. The values for the two other classes are calculated 5.56 and 9.88, respectively. The results show that heart perturbation of third classes is more than second and first classes (Figs. 5*a–c*). In other words, the heart works with more disorder compared with the two other classes. Also, the results reveal that lung of the unhealthy person acts with more disorder than the healthy person. According to (2), the healthy measure of respiration rate is obtained at 1.54, 1.9 and 2.3 for three classes in turn (Figs. 5*d–f*). The experiments were repeated for 29 other persons of every class. So, the borders of the healthy and the unhealthy cardiorespiratory system were determined to benefit the proposed methods [(2) than (3)] and shown in Fig. 6. Ultimately, the outcome results of the sensor reveal the proposed system is a good candidate for monitoring heartbeat and respiration rate as well as can be an appropriate device for diagnosis of the initial problems in the cardiorespiratory system.

## 5 Conclusions

In this Letter, a comprehensive description of developing a wireless sensor with features including compact, small and very light weight for measuring heartbeat and respiration rate based on motion sensors were presented. The developed sensor was tested in two scenarios and compared with a commercial ECG apparatus and also a belt around the abdomen as a gold standard. The outcome results of the scenarios reveal the sensor records heartbeat and respiration rate with RMSE <2.27 and 0.93 and also SD<1.26 and 0.63, respectively. Additionally, the results show that the sensor is able to identify the initial problems in the cardiorespiratory system. According to this, the sensor can be a good candidate for human vital sign monitoring for healthcare applications.
